# Vasculogenic Mimicry in Head and Neck Squamous Cell Carcinoma—Time to Take Notice

**DOI:** 10.3389/froh.2021.666895

**Published:** 2021-03-31

**Authors:** Abdelhakim Salem, Tuula Salo

**Affiliations:** ^1^Department of Oral and Maxillofacial Diseases, Clinicum, University of Helsinki, Helsinki, Finland; ^2^Translational Immunology Research Program (TRIMM), Research Program Unit, University of Helsinki, Helsinki, Finland; ^3^Cancer and Translational Medicine Research Unit, University of Oulu, Oulu, Finland; ^4^Helsinki University Hospital, Helsinki, Finland

**Keywords:** vasculogenic mimicry, tumor cell-lined vessels, head and neck squamous cell carcinoma, epithelial-mesenchymal transition, hypoxia, survival, prognosis, lymphatic mimicry

## Abstract

Head and neck squamous cell carcinoma (HNSCC) is a group of common cancers characterized by a swift growth pattern, early metastasis, and dismal 5-year survival rates. Despite the recent advances in cancer management, the multimodality approach is not effective in eradicating HNSCC. Moreover, the clinical response to the antiangiogenic therapy remains considerably limited in HNSCC patients, suggesting that tumor perfusion can take place through other non-angiogenic pathways. Tumor cell-induced angiogenesis is one of the main hallmarks of cancer. However, at the end of the previous millennium, a new paradigm of tumor cell-associated neovascularization has been reported in human melanoma cells. This new phenomenon, which was named “vasculogenic mimicry” or “vascular mimicry” (VM), describes the ability of aggressively growing tumor cells to form perfusable, matrix-rich, vessel-like networks in 3-dimensional matrices *in vitro*. Similar matrix-rich VM networks were also identified in tissue samples obtained from cancer patients. To date, myriad studies have reported intriguing features of VM in a wide variety of cancers including HNSCC. We aim in this mini-review to summarize the current evidence regarding the phenomenon of VM in HNSCC—from the available detection protocols and potentially involved mechanisms, to its prognostic value and the present limitations.

## Introduction

Head and neck squamous cell carcinoma (HNSCC) represents a group of common and deadly cancers that collectively account for more than 90% of all tumors arising in the head and neck region. It originates in the epithelial lining of the oral cavity, oropharynx, hypopharynx, and larynx [1–3]. Of these, oral squamous cell carcinoma (OSCC) is the most common subtype of HNSCC, where the majority of tumors are encountered in the anterior 2/3 of tongue (aka oral tongue, OTSCC) [4]. The incidence of HNSCC has recently been increasing in many regions of the world with a shifting trend toward women and younger populations [5, 6]. Indeed, chemical carcinogens such as tobacco- and alcohol-derived products are the major inducers of the tumorigenesis in HNSCC patients. Increasingly, infection with human papillomavirus (HPV), mainly by HPV-16 or HPV-18 types, is linked to HNSCC. However, HPV-positive tumors arise predominantly in the oropharyngeal region including tonsils, base of tongue and soft palate [6–8].

Generally, complete surgical resection of the tumor remains the primary approach for treating patients with HNSCC. Depending on the disease stage, surgery could be followed by adjuvant radiotherapy with or without chemotherapy. Unfortunately, such multimodality approach can severely impact the quality of patient's life, and it remains ineffective in a considerably high number of patients [6–9]. Recently, the U.S. Food and Drug Administration (FDA) has approved the use of two immune checkpoint inhibitors (i.e., Pembrolizumab and Nivolumab) for treating recurrent/metastatic HNSCC patients. However, the overall response rates of these immunotherapeutics were very limited ranging between 13 and 18% [10]. Despite the remarkable progress in cancer research and management, the 5-year survival rate of HNSCC patients remains dismal, emphasizing the urgent need to identify more effective therapies and clinically reliable biomarkers.

Cancer metastasis is one of the main hurdles for a successful therapy of cancer patients, accounting for the majority of cancer-specific deaths [11]. HNSCC is featured by high invasion rates and most patients present with regional metastasis to cervical lymph nodes at the time of diagnosis. Thereby, the involvement of regional lymph nodes is one of the most important prognostic parameters in OSCC, which can confer up to a 50% decrease in the survival outcomes [12, 13]. Angiogenesis is an essential process for tumor development and metastasis, which represents an attractive target in cancer therapy. HNSCC is rich in several angiogenesis-related factors, such as vascular endothelial growth factor (VEGF) [14]. However, monotherapy with anti-angiogenic agents has generally revealed a low or modest response in HNSCC patients, suggesting that tumor perfusion can take place independently from angiogenesis [14, 15].

**Figure 1:**
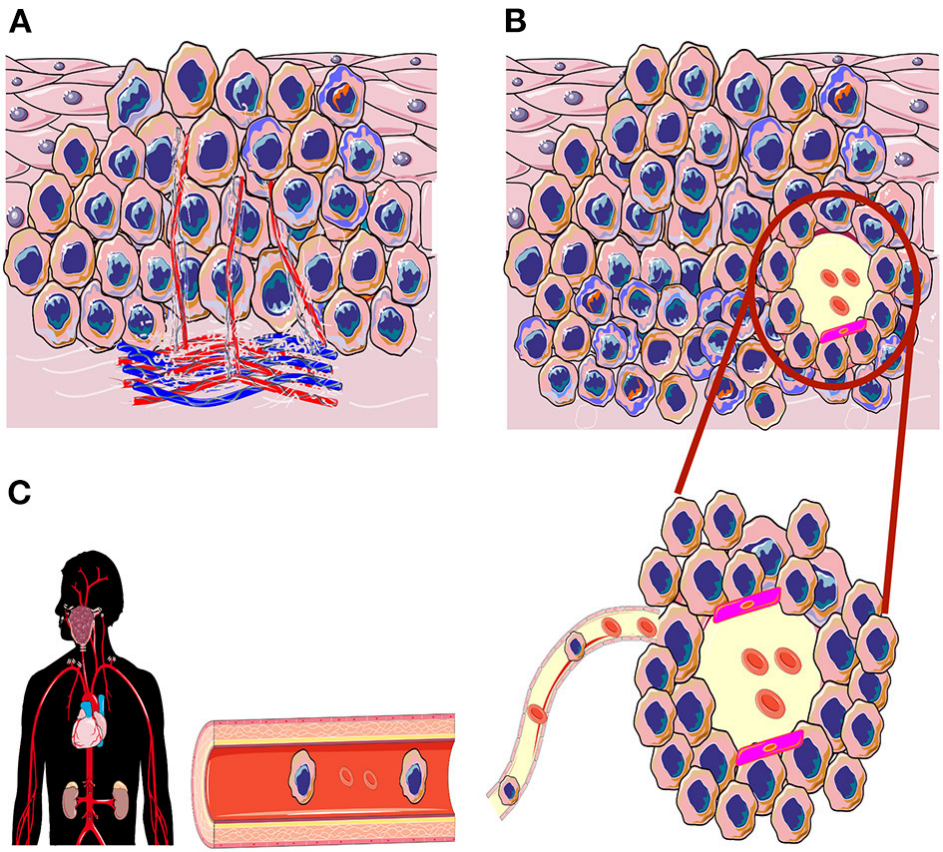
Schematic illustration of angiogenesis and vasculogenic mimicry (VM) in the tumorigenesis of head and neck squamous cell carcinoma. **(A)** In induced angiogenesis, tumor cells release potent pro-angiogenic factors that induce the growth of inter- and intratumoral vasculature to enrich the tumor microenvironment. **(B)** In the VM, the aggressively invading tumors can generate *de novo*, perfusable, and matrix-rich vessel-like channels as an alternative non-angiogenic neovascularization method. **(C)** These new tumor cell-lined channels may also express a considerable levels of endothelial cell markers (magenta color; mosaic pattern); VM channels may reach out to the host vasculature and increase nutrient retrieval to nourish the hypoxic tumor tissue. Ultimately, these VM channels could be utilized as a dissemination route to facilitate distant metastasis.

In 1999, a new tumor-related angiogenic paradigm was suggested by Maniotis and colleagues. This concept, which has been named “vasculogenic mimicry” (VM; also referred to as “vascular mimicry”) describes the formation of tumor-derived, matrix-rich, vessel-like networks by aggressive melanoma cells on a three-dimensional (3D) matrix *in vitro* [16, 17]. These tumor cell-formed channels may facilitate the dissemination of tumor cells into blood stream, and hence contribute to metastasis and the poor survival outcomes. To date, this phenomenon has ignited a vibrant debate among cancer researchers, which resulted in an impressive body of reports showing intriguing aspects of the VM in different types of cancer including HNSCC (Figure 1) [18]. Based on the growing interest in the VM as an attractive therapeutic and prognostic target in cancer, we aimed in this mini-review to summarize the current knowledge regarding VM in HNSCC.

## Characterization of VM in HNSCC

### The Current Practice for VM Identification, Limitations, and New Proposals

The VM structures are currently identified in patient samples as intratumoral red blood cell (RBC)-containing lumens that stain positively for periodic acid-schiff (PAS) in the absence of any endothelium-related proteins such as CD31 or CD34 [19]. However, among the limitations of this method is that PAS+ regions are not deemed sensitive to permit VM quantification, and may also represent extracellular components that are not necessarily relevant to the VM [20–22]. Moreover, one of the main challenges regarding VM identification is to ascertain that such “PAS+ regions” are truly functional vessels rather than tumor cell-derived aggregates of glycoproteins in the tumor microenvironment. Thus, Valdivia et al. concluded that when identifying VM, PAS+ staining alone should be interpreted with caution [22]. In other studies, pan-cytokeratin (CK), as a squamous epithelial cell marker, combined with CD31/CD34, were utilized for double-labeled immunohistochemistry to identify the CK-positive and CD31- or CD34-negative, RBC-containing, VM vessels in OSCC tissues [23, 24]. However, the precise nature of these “hot spots,” whether they represent true VM or other unrelated structures (e.g., necrotic tissues or regressed blood vessels), remains ambiguous [19].

Importantly, a mosaic pattern of VM has been described, in which the intratumoral VM lumens express both endothelial and tumor cell markers. Initially, the genesis of this pattern was inferred as connection between endothelial cells and tumor cells in blood vessel walls [25]. However, the molecular signature data of VM-forming tumor cells has later revealed an upregulated expression of genes associated with “stemness,” including endothelial cell-specific markers [26]. Apparently, this unique signature capacitates aggressive tumor cells to exhibit a remarkable degree of phenotype plasticity [17, 26]. Consistent with these findings, endothelial and mesenchymal cell-relevant markers such as CD31, VE-cadherin and vimentin were expressed by cultured HNSCC cell lines as well as in primary and metastatic tumor tissues [27]. Noteworthy, such co-localization mosaic pattern of tumor/mesenchymal markers, despite its promising prognostic value, has not been considered when identifying the VM in HNSCC, which entails the need for a standardized characterization protocol [19]. Additional but less conclusive criteria were also set for detecting the VM in HNSCC samples including the absence of intratumoral hemorrhage, necrosis, or perivascular inflammatory cell infiltrate in the hotspot area [28].

### VM Identification *in vitro*

The *in vitro* assays showed the ability of different HNSCC cells to form distinct “honeycomb-like” tubular networks, similar to those formed by human umbilical vein endothelial cells (HUVEC), when cells were cultured on Matrigel® [27, 29–31]. In contrast, normal human keratinocytes remained in a dispersed single cell aggregation when cultured in the same matrix. Likewise, tumor cells did not form any tubular networks when they grew in confluent monolayer cultures [27, 29]. Interestingly, collagen type XVI was able through the NC11 domain to trigger vasculogenic phenotype in 2D- and 3D OSCC cell culture conditions, implying a role of culture contents in VM formation [31]. Furthermore, we found that the capacity of tumor cells to generate such VM networks on 3D matrices is not akin among all HNSCC-derived cell lines. Intriguingly, the formation of tube-like structures *in vitro* was dependent on key factors including, inter alia, the metastatic/invasive potential of the cultured tumor cells [32]. In OTSCC, for instance, the low-invasive SCC-25 cells failed to generate consistent tubular networks on Matrigel® compared with the high-invasive HSC-3 cells, although both cell lines were cultured under the same conditions [32].

## Epithelial-Mesenchymal Transition and VM Formation

Growing evidences suggest that the development of VM is an intricate process involving various molecular mechanisms and signaling pathways [33]. Epithelial-mesenchymal transition (EMT) is an important developmental process whereby cells abandon their epithelial traits and acquire mesenchymal-like cell phenotype [34]. During EMT, cells lose their cell polarity, forfeit their epithelial markers such as E-cadherin, and gain mesenchymal characteristics such as N-cadherin, vimentin, and fibronectin [35]. Indeed, there is good evidence that EMT may promote tumor metastasis. In addition, EMT has been suggested as a possible mechanism in the formation of VM [34, 36]. It has been reported that siRNA knockdown of EPH receptor-A2 inhibited VM formation and tumor cell invasion by regulating the expression of EMT-related molecules in HNSCC cell lines [30]. In this study, inhibition of VM formation *in vitro* was associated with a significant reduction in the expression of EMT-relevant molecules such as Twist and vimentin and upregulation of E-cadherin, claudin-4, and desmoglein-3.

Transforming growth factor beta (TGF-β) signaling is one of the main inducers of EMT in cancer progression and metastasis [34]. Interestingly, the exposure of cultured OTSCC cells to TGF-β1 resulted in striking endothelial phenotypic modulations including “cadherin switching”—marked by a robust decrease in E-cadherin with a concurrent increase in VE-cadherin [27]. Authors concluded that such “endotheliod” phenotype possessed by HNSCC cells may induce VM formation and facilitate tumor progression and metastasis.

Tumor hypoxia, or a low intratumoral oxygenation, is a pivotal microenvironmental factor that has been associated with tumor dissemination and poor clinical outcomes in HNSCC. Hypoxia-inducible factor-1 alpha (HIF-1α), for instance, is a key transcriptional regulator in adaptive response to hypoxic tumor microenvironment, which represents an interesting cancer drug target [37, 38]. Importantly, HIF-1α has been shown to mediate VM formation through the regulation of EMT in epithelial ovarian cancer, where the VM-positive samples highly expressed EMT-associated molecules such as Twist1, Slug, and VE-cadherin [39]. However, to our knowledge, the role of hypoxia in the development of VM in HNSCC patients has not been investigated to date. This lack of evidence should be addressed in future studies, as HIF-1α represents a strong indicator of treatment failure and worse outcomes in HNSCC patients [37].

The EMT program has been shown to confer a strong stemness feature to cancer cells by upregulating the expression of the pro-angiogenic factor VEGF-A [35]. In agreement with these data, VEGF-A was recently found to mediate VM formation in nasopharyngeal carcinoma (NPC) cells, suggesting another connecting mechanism between EMT and VM formation [40]. In the same study, authors showed that VM formation was dependent on both VEGF-A and the Epstein-Barr virus (EBV)-encoded latent membrane protein 1 (LMP1)— a key effector of EBV-mediated B cell transformation. Of note, knockdown of LMP1 or VEGFR1 strongly interfered with the tubular structures during the tubulogenesis of VM *in vitro* [40]. These observations suggest that EMT-related mechanisms may underpin the development of VM in EBV-mediated NPC.

## Prognostic Value of VM in HNSCC

A growing body of evidence indicates that the presence of tumor cell-lined VM associates significantly with shorter survival outcomes of cancer patients [41]. Thus, several studies have investigated the feasibility to harness VM as a clinical prognosticator in patients with HNSCC [24, 28, 40, 42, 43]. Notably, all these studies concluded that positive VM status can predict shorter survival outcomes and dismal clinicopathological parameters in patients with HNSCC (Table 1). In OSCC, VM-positive status was positively correlated with more lymph node metastasis (LNM), TNM (tumor, node, and metastasis) staging, and larger tumor size [24, 28]. In line with these observations, two studies with 371 laryngeal SCC cases showed a significant correlation between VM and the advanced tumor stages (III and IV), LNM and TNM [42, 43]. Furthermore, Xu et al. analyzed the prognostic significance of VM formation on the progression-free survival of 40 patients with NPC. Interestingly, the VM-positive group had significantly shorter survival than the VM-negative group [40]. We showed in a recent meta-analysis study that positive-VM status was associated with poor overall survival (hazard ratio = 0.50; 95% confidence interval: 0.38–0.64) in patients with SCC of head and neck or esophagus, which remained consistent following the subgroup analysis of the studies [19].

**Table 1:** List of studies that investigated the prognostic significance of VM in HNSCC patients.

**Country**	**Tumor type**	**Tumor site**	**Sample size**	**VM+ cases**	**Main findings**	**References**
Taiwan	OSCC	Tongue, cheek, gingiva, floor of mouth, palate, lip	112	41 (36.60%)	Patients with more VM had poorer survival	[24]
China	OSCC	Tongue, gingiva, palate, tonsil	190	60 (31.57%)	VM predicted shorter survival	[28]
China	LSCC	Larynx	203	44 (21.67%)	VM predicted poor clinical parameters and shorter survival	[42]
China	LSCC	Larynx	168	37 (22.02%)	VM was shown as an independent prognostic factor for diseases-free survival	[43]
China	NPC	Nasopharynx	40	23 (57.50%)	VM predicted shorter survival	[40]

*HNSCC, Head and neck squamous cell carcinoma; OSCC, oral squamous cell carcinoma; LSCC, laryngeal squamous cell carcinoma; NPC, nasopharyngeal carcinoma; VM, vasculogenic mimicry*.

## Lymphatic Mimicry in HNSCC

Lymphatic metastasis represents the main route of tumor cell dissemination in HNSCC [13]. Very recently, we introduced a potential novel mechanism of lymphatic metastasis in HNSCC—lymphatic mimicry (LM). The LM describes a process whereby tumor cells form CK^+^/lymphatic vessel endothelial hyaluronic acid receptor 1 (LYVE-1)^+^ mosaic endothelial-like vessels in cancer tissues and *in vitro*. The multiplex immunostaining revealed that LM structures were negative for other lymphatic markers such as podoplanin. Interestingly, knockdown of LYVE-1 inhibited the capacity of tumor cells to generate consistent LM-vessels *in vitro*, and reduced tumor cell metastasis *in vivo* [32]. These important findings call for more studies to better define this novel, VM-related, phenomenon in the future, which could also provide avenues for developing anti-lymphangiogenic drugs in HNSCC.

## Concluding Remarks and Limitations

There are myriad studies that provided interesting mechanistic and functional insights into the concept of VM in many cancers including HNSCC. However, these studies have concurrently revealed the immense complexity of different overlapping signaling pathways in the tumor microenvironment and their plausible role in VM formation. Collectively, these findings illuminate the significance of investigating tumor cell plasticity in aggressive tumors such as HNSCC [17, 26, 33]. Therefore, in addition to the aforementioned traditional method, the mosaic tumor cell-formed lumens should be evaluated and considered as a competent way for identifying VM in cancer tissues. Also, owing to its key contribution to tumorigenesis and anticancer treatment failure, there is an urgent need to investigate the role of hypoxia in the development of VM in HNSCC.

In spite of the spirited debate ignited by this phenomenon, some concerns were raised concerning the existence of VM. These critics include, among others, whether it is possible to differentiate VM structures from the intratumoral endothelial vessels unambiguously?; and are these VM structures representing real vessels that contribute to blood flow? [26]. Moreover, the lack of *in vivo* evidence for functional VM channels, the current ambiguous methodological protocol, and the shortage of reliable biomarkers for identifying VM represent an unceasing challenge to date. Thus, more studies are still needed to better characterize and understand this intriguing phenomenon and to assess their full prognostic and therapeutic potential in HNSCC and in other recalcitrant tumors.

## Author Contributions

AS designed and wrote the manuscript. TS reviewed and edited the manuscript. Both authors contributed to the article and approved the submitted version.

## Conflict of Interest

The authors declare that the research was conducted in the absence of any commercial or financial relationships that could be construed as a potential conflict of interest.
